# Risk factors and outcomes associated with acute kidney injury following ruptured abdominal aortic aneurysm

**DOI:** 10.1186/1471-2369-14-99

**Published:** 2013-05-01

**Authors:** Ilana Kopolovic, Kim Simmonds, Shelley Duggan, Mark Ewanchuk, Daniel E Stollery, Sean M Bagshaw

**Affiliations:** 1 Division of Critical Care Medicine, Faculty of Medicine and Dentistry, University of Alberta, 3C1.12 Walter Mackenzie Centre, 8440-112 Street, Edmonton, Alberta T6G 2B7, Canada; 2 Infectious Disease Epidemiology, Surveillance and Assessment Branch, Community and Population Health Division, Alberta Health & Wellness, 23rd Floor, Telus Plaza NT 10025 Jasper Avenue, Edmonton, Alberta, T5J 1S6, Canada; 3 Grey Nuns Community Hospital, Division of Critical Care Medicine, Faculty of Medicine and Dentistry, University of Alberta, 100 Youville Dr W, Edmonton, Alberta T6L 5X8, Canada

## Abstract

**Background:**

Current data describing the epidemiology of acute kidney injury (AKI) following repair of ruptured abdominal aortic aneurysm (rAAA) are limited and long-term outcomes are largely unknown. Our objectives were to describe the incidence rate, risk factors, clinical course and long-term outcomes of AKI following rAAA repair.

**Methods:**

Retrospective population-based cohort study of all referrals undergoing emergency repair of rAAA in Northern Alberta from January 1, 2002 to December 31 2009. Demographic, clinical, physiologic and laboratory data were extracted. AKI was defined and classified according to the AKIN criteria.

**Results:**

In total, 140 patients survived to receive emergent rAAA repair. Post-operative AKI occurred in 75.7% of patients (n = 106), 78.3% (n = 83) of which occurred during the initial 24 hours of ICU admission. AKIN stage 1, 2, and 3 occurred in 47 (33.6%), 36 (25.7%) and 23 (16.4%), respectively, with 19 patients receiving renal replacement therapy (RRT). Several clinical and biochemical patient factors were associated with incident AKI, including baseline estimated glomerular filtration rate (eGFR) < 60 mL/min/1.73 m^2^ (odds ratio [OR] 2.94; 95% CI, 1.15-7.51, p = 0.03), need for mechanical ventilation (OR 22.7; 95% CI, 7.0-72.1, p < 0.0001) and vasoactive therapy (OR 9.9; 95% CI, 3.0-32.2, p < 0.0001) and higher mean APACHE II scores (25.7 [8.2] vs. 16.3 [4.9], p < 0.0001). AKI was associated with a higher ICU (28.3% vs. 0%; p = 0.0008) and in-hospital case-fatality rate (35.9% vs. 0%, p = 0.0001). Of 102 survivors to discharge, 65.7% (n = 67) recovered to baseline kidney function. In multivariable analysis, greater severity of AKI (OR 5.01; 95% CI, 2.34-10.7, p < 0.001) and lower baseline eGFR (OR 0.96; 95% CI, 0.93-0.99, p = 0.03) were associated with non-recovery. AKI remained independently associated with 1-year mortality after adjusting for age, sex, comorbidity, and illness severity (OR 5.21; 95% CI, 1.04-26.2, p = 0.045; AUC 0.83; H-L GoF, p = 0.26). Among survivors at 1-year, only 63.4% (n = 55) had complete kidney recovery.

**Conclusions:**

Following rAAA repair, AKI is a common complication independently associated with long-term post-operative mortality. A significant proportion of AKI sufferers in this setting fail to recover to baseline kidney function.

## Background

Rupture of abdominal aortic aneurysm (rAAA) is a relatively uncommon but devastating surgical emergency, occurring in an estimated 5.6 persons per 100,000 population and more common among men and older persons [1,2]. The vast majority of patients presenting with rAAA currently receive emergency open repair; however, the utilization of endovascular repair has increased [3]. The peri-operative morbidity and mortality associated with repair of rAAA continues to be burdensome. Short-term mortality has remained relatively stable at approximately 45-50% for open repair [4], and post-operative morbidity remains common, with reported rates of myocardial infarction and colonic ischemia of 24% and 9% respectively, and substantial rates of prolonged post-operative ventilatory and vasoactive support [5,6].

In addition, acute kidney injury (AKI) remains a common and important post-operative complication for patient receiving either elective or emergent AAA repair, occurring in 15-22% [7-9]. In rAAA, severe AKI requiring initiation of renal replacement therapy (RRT) has been described in approximately 4-24% of cases [9,10]. Few data have explored factors predictive of early post-operative AKI specifically in rAAA. In small cohort studies that have generally included both elective and ruptured AAA patients, older age, pre-morbid chronic kidney disease (CKD), intra-operative hypotension and low-cardiac output, prolonged ischemic time, receipt of peri-operative blood transfusion and post-operative rhabdomyolysis independently predicted post-operative AKI [7-9].

Post-operative AKI has been associated with less favorable outcomes following rAAA repair, including higher risk of death and prolonged duration of hospitalization [9]. The modifying impact of post-operative AKI on outcomes may be under-appreciated in the setting of emergency procedures, such as for rAAA [11]. Available data for rAAA repair complicated by AKI have described peri-operative mortality rates ranging 53-69% [12-14]. Importantly, very little is known about kidney recovery in those suffering AKI following rAAA repair [9]. This is a concerning knowledge gap, as recent data imply a single episode of AKI independently predicts both long-term declines in survival and risk for incident or progressive CKD [15-17]. To date, no studies that have evaluated the long-term survival and kidney outcomes following rAAA repair complicated by AKI.

Accordingly, our objectives were to describe, in a population-based cohort of patients surviving repair of rAAA (a) the incidence and spectrum in severity of AKI, including utilization of RRT; (b) the clinical and operative factors associated with post-operative AKI; and (c) the short and long-term survival and kidney recovery associated post-operative AKI.

## Methods

The study protocol was approved by the Health Research Ethics Board at the University of Alberta prior to commencement. The requirement for written consent was waived. The reporting of this study follows the STROBE guideline [18].

### Design, setting and participants

This was a retrospective population-based cohort study. The study was performed in the province of Alberta, Canada (population ~ 3.7 million in 2009). In Alberta, there are eight geographical health zones and two regional referral centers for all major vascular surgery. In the five health zones comprising central and northern Alberta (population ~ 2.0 million), all major vascular surgery and all cases of rAAA surviving to hospital are referred to the Grey Nuns Community Hospital (GNH). For the purpose of establishing population incidences, patient residency in this region was confirmed by verification of postal codes. The GNH is a university-affiliated, 347-bed hospital in located in Edmonton, Alberta. The GNH intensive care unit (ICU) is a closed, eight bed mixed medical-surgical unit staffed by dedicated intensivists and receives ~500 admissions annually. All patients with emergent repair of rAAA are monitored and supported in the ICU post-operatively. All adult (≥18 year) patients admitted to the GNH with a primary diagnosis of rAAA undergoing surgical repair during the study period (January 2002 to December 2009 inclusive) were eligible for inclusion; those with a pre-morbid diagnosis of end stage kidney disease (ESKD) were excluded.

### Study protocol

Patients were identified by interrogation of an ICU-specific clinical/administrative database (Minimal Data Set [MDS] database) between January 1, 2002 and December 31, 2009 [19]. The MDS database is maintained by the regional Division of Critical Care Medicine, and routinely captures demographic, clinical, physiologic and outcome data on all admissions to the five general medical/surgical intensive care units in Edmonton Zone. We extracted data on patient demographics, ICU admission source, ICU admission date, post-operative status, co-morbid conditions, necessity for mechanical ventilation, vasoactive support, Acute Physiology and Chronic Health Evaluation (APACHE) II score [20], ICU and hospital duration of stay and ICU, in-hospital and minimum 1-year mortality. Individual patient medical records, including those of the index hospitalization as well as previous and post-discharge visits in survivors, were also interrogated for additional clinical, pharmacologic (i.e. pre-admission prescription medications), physiologic and laboratory data, along with operative/anesthesia records (i.e. procedure duration, blood loss, and transfusions).

### Operational definitions

Patients were said to have a history of *hypertension, coronary artery disease (CAD), stroke* or *smoking* if these were documented in their medical record. Presence of *diabetes mellitus (DM)* was defined by prior documentation in the medical record or by an admission HbA1c ≥6.5%. Urine output data were available for the 24 hours after ICU admission. Oliguria was defined as a urine output < 400 mL/24 hour. cTnI was measured using the Beckman-coulter assay which detects the presence of cTnI at a threshold concentration of 0.15 mcg/L [21]. Any cTnI ≥0.15 mcg/L was designated a positive result (TnI+). Baseline serum creatinine was the value documented in the 6-months prior to the index hospital admission and was available for all included patients. Baseline estimated glomerular filtration rate (eGFR) was calculated using the CKD-EPI equation [22].

*Acute kidney injury (AKI)* occurring in association with rAAA was defined according to the serum creatinine-based criteria of the Acute Kidney Injury Network (AKIN) classification system [23]. The diagnosis of AKI was confirmed for any patient with a >26.5 μmol/L increment in serum creatinine within a 48 hour period following presentation or a 1.5 fold increment in serum creatinine relative to the pre-hospital baseline creatinine value. Peak post-operative creatinine relative to pre-hospital baseline creatinine was used to assign severity stage of AKI. *Complete renal recovery* was defined as return to within 10% of baseline creatinine. *Non-recovery* was defined as < 10% decline from peak serum creatinine following AKI and/or RRT dependence at the time of death or hospital discharge. *Partial recovery* was defined as a reduction of >10% in serum creatinine from peak value, but with a final value >10% above baseline. Renal recovery, i.e., complete, partial, or none, was assessed at time of hospital discharge or death for hospital non-survivors. For those discharged without complete recovery of baseline renal function, renal recovery was re-evaluated at one-year following hospital discharge.

### Statistical analyses

Clinical variables and univariate comparison between groups are reported for normally or near normally distributed variables as means with standard deviations (SD) and compared by Student’s *t*-test or analysis of variance (ANOVA) and for non-normally distributed continuous data as medians with inter-quartile ranges (IQR) and compared by Mann Whitney *U* test or Kruskal Wallis test, respectively. Categorical data are reported as proportions and compared using Fisher’s Exact Test. Customized, multiple variable logistic regression models were created, using mortality or renal recovery as dependent variables, that considered age, sex, pre-existing chronic kidney disease, APACHE II score; blood loss/blood transfusion, mechanical ventilation, vasopressor use, renal replacement therapy (RRT) and post-operative AKI as covariates. Data are reported as odds ratios (OR) with 95% confidence intervals (CI). Model calibration and fit were assessed by the area under the receiver operating characteristic curve (AUC) and the Hosmer-Lemeshow goodness of fit (GoF) test, respectively. Crude survival stratified by study group was assessed graphically by the Kaplan-Meier product limit estimator and compared with the log-rank test. A p-value of < 0.05 was considered statistically significant for all comparisons. Analysis was performed using Intercooled Stata Release 10 (Stata Corp, College Station, TX).

## Results

### Epidemiology and patient characteristics

In total, 142 patients presented with a primary diagnosis of rAAA and received operative repair during the study period, of which, two were excluded due to pre-morbid ESKD. This represented a population-based incidence of 11.9 events per million population per year (95% CI, 9.9-14.1; n = 133; n = 7 excluded due to primary postal address located outside the defined geographic catchment). The incidence of rAAA increased by an estimated 6.9% per million year (95% CI, 5.9-13.2, p = 0.04) over the study period.

Of these, 96.4% (n = 135) received open operative repair with the remaining undergoing endovascular repair. Among all patients (n = 140), mean (SD) age was 71.1 (8.8) years, 85.0% (n = 119) were male, 70.7% (n = 99) had hypertension and 91.1% (n = 113) had a smoking history. Mean (SD) pre-morbid eGFR was 65.8 (19.9) mL/min/1.73 m^2^ and 33.6% (n = 47) had an eGFR < 60 mL/min/1.73 m^2^ at baseline (Table 1).

**Table 1:** Summary of baseline characteristics, intra-operative parameters and acute physiologic stratified by AKI status

**Parameter**	**Total**	**AKI**	**No AKI**	**p**
n, %	140	106 (75.7)	34 (24.3)	-
Age (years) [mean [SD])	71.1 (8.8)	71.6 (8.0)	69.7 (10.9)	0.27
Male Sex (n, %)	119 (85.0)	88 (83.0)	31 (91.2)	0.41
**Co-morbid disease**				
Hypertension (n, %)	99 (70.7)	77 (72.6)	22 (64.7)	0.39
Coronary artery disease (n, %)	50 (35.2)	37 (35.2)	13 (39.4)	0.68
Stroke (n, %)	14 (10.1)	10 (9.5)	4 (11.8)	0.75
Diabetes Mellitus (n, %)	24 (17.1)	20 (18.9)	4 (11.8)	0.44
Ever smoked (n, %)	113 (91.1)	82 (90.1)	31 (93.9)	0.73
Baseline eGFR (mean [SD])	65.8 (19.9)	63.1 (20.1)	74.1 (17.0)	0.005
Baseline eGFR < 60 mL/min/1.73 m^2^ (n, %)	47 (33.6)	41 (38.7)	6 (17.7)	0.04
**Pre-operative Medications**				
HMG-CoA Reductase Inhibitor (n, %)	42 (31.1)	33 (32.4)	9 (27.3)	0.67
ACE inhibitor/ARB (n, %)	46 (34.1)	35 (34.1)	11 (33.3)	1.0
Beta-blocker (n, %)	39 (28.9)	26 (25.5)	13 (39.4)	0.18
**Intra-operative variables**				
Operative Duration (min) (mean [SD])	228 (74)	221 (69)	261 (92)	0.03
Supra-renal Aortic Clamp (n, %)	22 (16.7)	17 (17.0)	5 (15.6)	1.0
Estimated blood loss (L) (mean [SD])	3.4 (3.2)	3.9 (3.6)	1.8 (1.4)	0.002
Red cell transfusion (units) (median [IQR])	7 (3–12)	9 (5–14)	2 (0–4)	< 0.0001
**Post-operative variables**				
Mechanical ventilation (n, %)	120 (85.7)	102 (96.2)	18 (52.9)	< 0.001
Vasoactive support (n, %)	54 (40.6)	51 (50.5)	3 (9.4)	< 0.001
APACHE II score (mean [SD])	23.4 (8.5)	25.7 (8.2)	16.3 (4.9)	< 0.001
MAP (mm Hg) (mean [SD])	63.4 (13.8)	62.5 (14.6)	66.1 (11.3)	0.18
Heart rate (beats/min) (mean [SD])	111 (21)	113 (21)	103 (15)	0.01
PaO_2_ (cm H_2_O) (mean [SD])	72.9 (28.4)	73.2 (31.5)	72.0 (15.5)	0.82
PaCO_2_ (cm H_2_O) (mean [SD])	41.9 (7.2)	42.5 (7.6)	39.9 (5.6)	0.06
pH (mean [SD])	7.26 (0.10)	7.24 (0.10)	7.31 (0.07)	0.002
Hematocrit (mean [SD])	0.276 (0.05)	0.267 (0.05)	0.305 (0.04)	0.001
White cell count (cells/10^9^) (mean [SD])	12.8 (4.4)	12.8 (4.5)	12.9 (4.3)	0.92
Serum sodium (mmol/L) (mean [SD])	142 (4.0)	143 (3.6)	140 (3.0)	< 0.001
Serum potassium (mmol/L) (mean [SD])	4.9 (0.9)	5.0 (0.9)	4.5 (0.5)	0.001
Serum glucose (mmol/L) (mean [SD])	10.1 (2.9)	10.4 (2.9)	9.1 (2.2)	0.01
Serum troponin-I elevation (n, %)	78 (55.7)	69 (65.1)	9 (26.5)	< 0.001

Abbreviations: *APACHE II* = Acute Physiology and Chronic Health Evaluation; *eGFR* = estimated glomerular filtration rate; *ACE* = angiotensin converting enzyme; *ARB* = angiotensin receptor blocker; *MAP* = mean arterial pressure.

### Incidence and peri-operative factors associated with AKI

The cumulative incidence of AKI, defined by the AKIN criteria, was 75.7% (n = 106). Of these, 78.3% (n = 83) fulfilled AKIN criteria within 24 hours after ICU admission. AKIN stage 1, 2 and 3 occurred in 47 (33.6%), 36 (25.7%) and 23 (16.4%), respectively. The median (IQR) time to maximum AKIN stage was 1 day (0–5). RRT was received by 19 patients (13.6%). Of these, the majority (n = 13, 56.5%) achieved AKIN stage 3 (Tables 1 and 2).

**Table 2:** Summary of kidney function and outcomes stratified by severity of AKI

	**All**	**No AKI**	**AKIN stage 1**	**AKIN stage 2**	**AKIN stage 3**	**P**^**¶**^
	**(n = 140)**	**(n = 34)**	**(n = 47)**	**(n = 36)**	**(n = 23)**	
Baseline SCr (μmol/L) (median [IQR])	92 (81–110)	89 (80–103)	98 (83–118)	87 (80–112)	100 (85–124)	0.12
Baseline eGFR < 60 mL/min/1.73 m^2^ (no, %)	50 (35.5)	11 (26.2)	20 (42.6)	12 (38.7)	7 (33.3)	0.424
Baseline eGFR (mL/min/1.73 m^2^) (mean [SD])	65 (21)	71 (19)	60 (22)	67 (21)	63 (18)	0.078
Urine output (24 hr) (mL) (mean [SD])	1642 (1165)	1970 (852)	1809 (1315)	1357 (1234)	1266 (978)	0.04
Oliguria (< 400 mL/24 hr) (n, %)	16 (11.5)	0 (0)	3 (6.5)	8 (22.2)	5 (21.7)	0.003
Peak SCr (μmol/L) (median [IQR])	169 (123–277)	102 (90–122)	155 (135–190)	219 (175–262)	460 (358–460)	0.0001
Delta SCr (baseline to peak) (μmol/L) (median [IQR])	77 (31–139)	15 (4–22)	55 (42–80)	127 (94–148)	370 (278–464)	0.0001
Time to Peak SCr (d) (median [IQR])	1 (0–5)	-	1 (0–2)	1 (0–3)	6 (2–15)	0.0001
RRT (n, %)	19 (13.6)	0 (0)	2 (4.3)	4 (11.1)	13 (56.5)	< 0.001
Discharge SCr (μmol/L) (median [IQR])	107 (83–184)	82 (73–90)	108 (86–108)	112 (83–194)	244 (148–412)	0.0001
Discharge SCr* (μmol/L) (median [IQR])	87 (79–112)	82 (73–90)	96 (85–112)	85 (73–112)	168 (116–278)	0.0001
Delta SCr (baseline to discharge)* (μmol/L) (median [IQR])	1 (−9 – 16)	6 (−9 – 13)	0 (−2 – 26)	24 (0–96)	92 (42–189)	0.0001
RRT at hospital discharge (n, %)	4 (17.4)	0 (0)	0 (0)	0 (0)	4 (17.4)	0.001
ICU Mortality (n, %)	30 (21.4)	0 (0)	11 (23.4)	12 (33.3)	7 (30.4)	< 0.001
Hospital Mortality (n, %)	38 (27.1)	0 (0)	13 (27.6)	14 (38.9)	11 (47.8)	< 0.001
ICU stay (d) (median [IQR])	4 (2–9)	3 (2–5)	3 (1–7)	4 (2–10)	14 (7–23)	< 0.001
Hospital stay (d) (median [IQR])	11 (7–19)	9 (7–11)	10 (7–16)	11 (3–24)	23 (13–32)	< 0.001

Abbreviations: *SCr* = serum creatinine; *eGFR* = estimated glomerular filtration rate; *RRT* = renal replacement therapy.

*Survivors and RRT independent.

^¶^ P-value for comparison across groups.

Several factors were found associated with post-operative AKI including a baseline eGFR < 60 mL/min/1.73 m^2^ (odds ratio [OR] 2.94; 95% CI, 1.15-7.51, p = 0.03), greater intra-operative blood loss (3.9 [3.6] vs. 1.8 [1.4] L, p = 0.002), and greater number of units of transfused red cells (9 [5–14] vs. 2 [0–4] units, p < 0.001). In addition, patients developing AKI were more likely to be supported with mechanical ventilation (OR 22.7; 95% CI, 7.0-72.1, p < 0.0001), vasoactive therapy (OR 9.9; 95% CI, 3.0-32.2, p < 0.0001) and had higher illness severity scores (p < 0.0001). Post-operatively, AKI patients had lower serum pH (p = 0.002), lower serum hematocrit (p = 0.001), higher serum sodium (p < 0.001), higher serum potassium (p = 0.001), and were more likely to have elevated cardiac-specific troponin-I values (p < 0.001) (Table 1).

Several trends were apparent in stratifying patients by severity of kidney injury (Table 2). With more severe AKI, the proportion of patients with a detectable cTnI increased (p = 0.05), as did the likelihood of receiving vasoactive support (p = 0.002) and RRT (p = 0.001).

### Hospital outcomes

**Figure 1:**
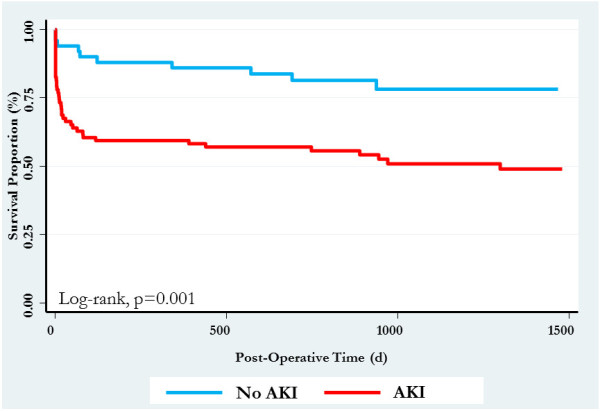
Kaplan-Meier survival curve stratified by post-operative AKI.

Overall, crude ICU and in-hospital mortality were 21.4% (n = 30) and 27.1% (n = 38), respectively. AKI was associated with a higher unadjusted ICU (28.3% vs. 0%; p = 0.0008) and in-hospital case-fatality rate (35.9% vs. 0%, p = 0.0001), compared to those with no AKI. In addition, post-operative AKI, stratified by severity, was associated with a dose–response increase in duration of ICU and hospital stay (Table 2). The unadjusted long-term survival stratified by post-operative AKI is shown in Figure 1.

### Renal recovery

**Figure 2:**
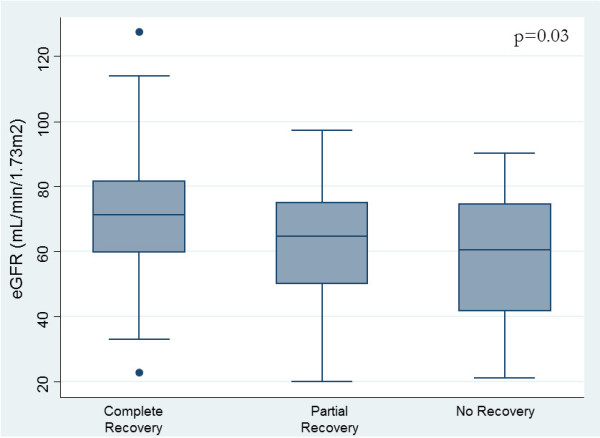
Renal recovery status at hospital discharge stratified by baseline estimated glomerular filtration rate.

Of 102 (72.9%) survivors to hospital discharge, the majority had complete recovery of kidney function (Table 3). Factors associated with partial or non-recovery of kidney function included greater illness severity (p = 0.005), lower pre-morbid eGFR (p = 0.03), greater severity of AKI (p < 0.001), and longer duration to peak serum creatinine (p < 0.001) (Figure 2). In multivariable analysis, only greater severity of AKI (OR 5.01; 95% CI, 2.34-10.7, p < 0.001) and lower baseline eGFR (OR 0.96; 95% CI, 0.93-0.99, p = 0.03) were independently associated with partial or non-recovery of function, after adjustment for demographics, comorbidities, and illness severity (AUC 0.85; H-L GoF, p = 0.28).

**Table 3:** Risk factors for non-recovery of kidney function at hospital discharge or death among patients with AKI

	**All patients (n = 102)**	**Complete RR (n = 67)**	**Partial RR (n = 26)**	**Non-recovery (n = 9)**	**P**^**¶**^
Age (years) (mean [SD])	69.9 (9.2)	69.8 (9.9)	69.1 (7.7)	73.7 (7.3)	0.43
APACHE II score (mean [SD])	20.4 (6.3)	19.0 (5.9)	23.7 (6.5)	21.1 (5.5)	0.005
Stage 3 AKI (no, %)	12 (11.8)	0 (0)	6 (23.1)	6 (66.7)	< 0.001
Male Sex (no, %)	87 (85.3)	60 (89.6)	21 (80.8)	6 (66.7)	0.10
Hypertension (no, %)	71 (69.6)	46 (68.6)	17 (65.4)	8 (88.9)	0.41
CAD (no, %)	37 (37)	21 (32.3)	13 (50.0)	3 (33.3)	0.30
Diabetes Mellitus (no, %)	13 (12.8)	9 (13.4)	3 (11.54)	1 (11.1)	1.0
Pre-op statin (no, %)	28 (28.0)	15 (23.1)	12 (46.2)	1 (11.1)	0.049
Pre-op ACEI (no, %)	30 (30.0)	17 (26.2)	11 (42.3)	2 (22.2)	0.30
Pre-op BB (no, %)	30 (30.0)	19 (29.2)	9 (34.6)	2 (22.2)	0.79
Baseline eGFR (mL/min/1.73 m^2^) (mean [SD])	70.6 (17.6)	73.6 (17.3)	66.6 (15.6)	59.5 (20.8)	0.03
Baseline eGFR < 60 mL/min/1.73 m^2^ (no, %)	25 (24.1)	14 (20.9)	6 (23.1)	5 (55.6)	0.09
Vasopressors in ICU (no, %)	25 (25.8)	15 (23.8)	5 (20.0)	5 (55.6)	0.12
Troponin (+) ve (no, %)	42 (65.6)	20 (62.5)	18 (78.3)	4 (44.4)	0.16
Time to peak SCr (day) (median [IQR])	1 (0–5)	0 (0–1)	3 (1–6)	5 (5–13)	< 0.001
OR duration (min) (median [IQR])	225 (180–285)	225 (173–270)	210 (180–285)	240 (180–285)	0.85
RRT (no, %)	8 (7.8)	1 (1.5)	3 (11.5)	4 (44.4)	< 0.001

Abbreviations: *AKI* = acute kidney injury; *CAD* = coronary artery disease; *eGFR* = estimated glomerular filtration rate; *OR* = operating room; *RRT* = renal replacement therapy.

^¶^ P-value for comparison across groups.

### Long-term outcome

At 1-year, cumulative mortality was 33.6% (n = 47/140). Post-operative AKI was associated with higher 1-year mortality (42.3% vs. 5.9%; OR 11.8; 95% CI, 3.0 - ∞, p < 0.0001). In multi-variable analysis, AKI remained independently associated with 1-year mortality (OR 5.21; 95% CI, 1.04-26.2, p = 0.045; AUC 0.83; H-L GoF, p = 0.26) after adjusting for age, sex, comorbidity, and illness severity. Of survivors, 63.4% (n = 55) had complete recovery, 27.9% (n = 26) had partial recovery, and 8.6% (n = 8) patients had non-recovery of function.

## Discussion

We performed a population-based cohort study of all patients in a large geographic region surviving rAAA to receive operative repair to describe the incidence, risk factors and associated mortality and renal recovery of post-operative AKI.

### Key findings

We found the incidence of rAAA in Northern Alberta was approximately 11.9 per million population per year, which increased significantly during the study by 6.9% per million per year. We found the cumulative incidence of AKI to be high at 75.7%, with the majority of episodes occurring within the first 1–2 days of surgery. Of these, 16.4% achieved AKIN Stage 3 and 13.6% received RRT. Post-operative AKI was associated with several factors including illness severity, intra-operative blood loss, and greater intensity of post-operative support (i.e. mechanical ventilation, vasoactive therapy). We also found both short-term and long-term mortality and resource utilization were greater for with post-operative AKI. Despite this, we also found that among survivors, most completely recovered kidney function. Those not recovering were more likely to have more severe AKI and impaired baseline kidney function.

### Interpretation and context with prior literature

The post-operative incidence of AKI following rAAA has been reported to range between 20-46%; however, these rates are often defined in small cohorts of elective or mixed elective/emergency patients [10,12-14,24]. In addition, an accurate estimate of the burden of post-operative AKI has been hindered by the variable definitions applied to diagnosis AKI. Many studies have defined AKI simply by post-operative receipt of RRT, utilization of ICD-9 codes and/or various changes or thresholds in post-operative serum creatinine [7,9,10,14]. The use of post-operative RRT will better define the health resource utilization associated with more severe forms of AKI; however, this approach remains susceptible to bias due to the inconsistent thresholds and practice variation for when and if to start RRT [25]. We believe our study is the largest to explore the incidence of post-operative AKI in patients receiving operative repair for rAAA. Using the AKIN criteria, we found a much higher incidence of AKI among rAAA patients than previously reported.

Prior literature has characterized a number of associative factors for post-operative AKI in elective AAA repair including: pre-morbid chronic kidney disease, procedural duration and kidney ischemic time; intra-operative hypotension, blood transfusion, rhabdomyolysis, low cardiac output syndrome, and post-operative treatment intensity (i.e. vasoactive therapy) [7,8,26]. Our results confirm these prior findings, showing longer operative duration, greater intra-operative blood loss and illness severity portend an increased risk for post-operative AKI.

Several studies have suggested less favorable outcomes for patients undergoing aortic aneurysm repair whose post-operative course is complicated by AKI, in particular when requiring RRT, with mortality rates in excess of 60% [7,8,12,14]. Recent data have also suggested even mild AKI not requiring RRT portends an increased risk for post-operative mortality [26]. We confirmed that AKI was associated with a dose–response increase in risk for death that persisted beyond index hospitalization among survivors.

It has been demonstrated that even non-RRT-dependent AKI influences long-term health outcomes, conferring increased risks of renal and non-renal outcomes, including mortality [17,27,28]. The long-term impact of AKI on renal function was recently demonstrated in a large cohort of patients undergoing major vascular surgery, which found that both transient and persistent renal dysfunction in the peri-operative period were independent predictors of the development of CKD [29]. There is limited data on renal recovery after AKI following rAAA, and much of it has defined “recovery” as RRT- independence, an insensitive definition. Residual renal insufficiency following episodes of RRT-dependent AKI in this setting has been reported in small cohorts [10,12]. Consistent with this, our data highlight that a proportion of patients with peri-operative AKI develop persistent kidney dysfunction following the event. Similar to our findings, others have described a correlation between severity of AKI, gauged by the degree of rise in serum creatinine, and failure to recover baseline kidney function or progression to CKD [15,30]. Recent literature has suggested that duration of AKI may be an even stronger prognosticator than severity [30,31], although we did not find a later peak in serum creatinine to be independently associated with renal outcomes.

### Limitations

Our study has several noteworthy limitations. First, we omitted patients with rAAA who died prior to presenting to hospital or were not referred for surgery. This may have unduly influenced our incidence estimate. Second, considering that rAAA is relatively uncommon, our cohort was small and as such, our analyses are subject to limited statistical power and errors of chance. However, our study is strengthened by a population-based cohort design. Third, we did not have detailed data on the duration of time from diagnosis to operative repair and recognize this may represent an important confounding factor for the association of AKI, intra-operative variables and mortality. Fourth, in our application of the AKIN criteria for AKI, we used the serum creatinine criteria only, due to urine output beyond the first 24 hours being variably documented. This may have also unduly influenced our incidence estimates for AKI. Fifth, we recognize the sole use of serum-creatinine based definitions for renal recovery may misclassify complete or partial recovery in patients with complicated post-operative courses, where suboptimal nutrition and loss of lean muscle mass may undermine creatinine as an accurate measure of GFR. Finally, the utilization of RRT was not necessarily standardized and clinical judgment and practice may have varied between clinicians.

## Conclusions

In summary, we present a population-based cohort of rAAA patients surviving to hospital to undergo operative repair, found a high incidence of post-operative AKI, and demonstrated its association with less favorable post-operative outcomes. Our data provide important information for prognostication and insight into factors associated with this clinical event. Importantly, a proportion of AKI sufferers in our cohort failed to recover baseline renal function, both at the time of hospital discharge and at one year following the event, indicating lasting morbidity. As the population of survivors of rAAA grows, our findings both inform those involved in its acute management, and imply the importance of appropriate follow-up to address long-term health consequences of the event.

## Competing interests

The authors declare that they have no competing interest.

## Authors’ contributions

SMB and IK conceived of the study. SMB, DES, SD, ME, and IK contributed to study design, including formulating and the research questions and ensuring a design to answer these. SMB, DES, SD, and ME established access to data. KS and SMB conducted the statistical analysis. The manuscript was drafted by IK and SMB and its methods and intellectual content were reviewed by KS, DES, SD, and ME who each made revisions. All authors provided their approval of the final version of the manuscript.

## Authors’ information

SMB is a critical care specialist attending general medical-surgical intensive care and cardiovascular surgical intensive care units at the University of Alberta Hospital, and an associate professor and clinician scientist at the University of Alberta. SD, DES, and ME are critical care specialists attending the intensive care unit at the Grey Nuns community Hospital. KS is an Epidemiologist for Alberta Health Services. IK is medical resident at the University of Alberta.

## Pre-publication history

The pre-publication history for this paper can be accessed here:

http://www.biomedcentral.com/1471-2369/14/99/prepub
